# Supplementary material for: Tunable magnonic crystal in a hybrid superconductor–ferrimagnet nanostructure
Source: Sci Rep. 2024 Oct 26;14:25594. doi: 10.1038/s41598-024-75492-0 (PMC11513118; doi:10.1038/s41598-024-75492-0)
Supplement: Supplementary file 1 — Supplementary Information. [file 41598_2024_75492_MOESM1_ESM.pdf]

Tunable magnonic crystal in a hybrid superconductor–ferrimagnet nanostructure

Julia Kharlan[1,2], Krzysztof Szulc[1,3], Jarosław W. Kłos[1], and Grzegorz Centała[1,*]

[1]ISQI, Faculty of Physics and Astronomy, Adam Mickiewicz University, Poznań, Poland
[2]Institute of Magnetism NASU and MESU, Kyiv, Ukraine
[3]Institute of Molecular Physics, Polish Academy of Sciences, Poznań, Poland
[*]grzcen@amu.edu.pl

1 Plane-wave method

In our studies, we do not consider the perpendicular standing SW modes, which have much higher frequencies. Therefore, for thin FM films, the SW amplitude can be assumed to be uniform across the thickness of the film: $m_{k,\alpha}(x,y) \approx m_{k,\alpha}(x)$, where $\alpha = x,z$.

The amplitude of SWs propagating in MC has a form of Bloch functions: $m_{k,\alpha}(x) = u_{k,\alpha}(x)e^{ikx}$. The periodic component of the Bloch function $u_{k,\alpha}(x)$ can be expanded in the Fourier series, and then the Bloch function can be written as

$$m_{k,\alpha}(x) = \sum_G m_{k,\alpha,G} e^{i(k+G)x}, \tag{S1}$$

where $G = 2\pi n/a$ is a reciprocal-lattice vector, indexed by integer $n = 0, \pm 1, \pm 2, \ldots$. The symbols $m_{\alpha,k,G}$ are the coefficients of the Fourier series of $u_{k,\alpha}(x)$. The stray field $H_{\rm sc}(x)$, as a periodic function, can also be expanded in the Fourier series:

$$H_{\rm sc}(x) = \sum_G H_{{\rm sc},G} e^{iGx}, \tag{S2}$$

where $H_{{\rm sc},G}$ are the coefficients of this expansion.

Dynamic demagnetizing field $\mathbf{h}_{\rm d}(x,y)$ is dependent on the spatial distribution of dynamic magnetization Eq. (7). For planar MC, the demagnetizing field can be expressed in terms of the Fourier coefficients for magnetization distribution[1]. Adopting the approach of[1] to a dynamical case, we obtain the following relations:

$$\begin{aligned} h_{{\rm d},x}(x,y) &= -\sum_G m_{k,x,G} e^{i(G+k)x} A(y, G+k), \\ h_{{\rm d},z}(x,y) &= 0. \end{aligned} \tag{S3}$$

The function $A(y,\kappa)$, which appears in (S3), has the form:

$$A(y,\kappa) = 1 - \frac{\cosh(|\kappa|(y-y_0))}{\cosh\left(\frac{|\kappa|d}{2}\right) + \sinh\left(\frac{|\kappa|d}{2}\right)}, \tag{S4}$$

where $y_0 = s - \frac{t+d}{2}$ is the center of FM layer. Since the FM layer is thin and $h_{{\rm d},x}(x,y)$ does not change significantly across the film thickness, we can take its value from the center of the layer, i.e. assume that $h_{{\rm d},x}(x) = h_{d,x}(x, y = y_0)$.

Substituting the Fourier expansions (S1-S3) into Eq. (8) leads to algebraic eigenvalue problem Eq. (9) where the elements of $\bar{\bar{\mathbf{M}}}_{xz}$ and $\bar{\bar{\mathbf{M}}}_{zx}$ have the following form:

$$\begin{aligned} M_{xz,G,G'} &= -\,\delta_{G,G'}\left(1 + \lambda_{\rm ex}^2 (G+k)^2 \frac{M_{\rm s}}{\tilde{H}_0}\right) - \frac{H_{{\rm sc},G'-G}}{\tilde{H}_0}, \\ M_{zx,G,G'} &= \;\delta_{G,G'}\left(1 + \left(\lambda_{\rm ex}^2 (G+k)^2 + A(y_0, G+k)\right)\frac{M_{\rm s}}{\tilde{H}_0}\right) + \frac{H_{{\rm sc},G'-G}}{\tilde{H}_0}, \end{aligned} \tag{S5}$$

where $\delta_{G,G'}$ is the Kronecker delta.

To solve the eigenproblem Eq. (9) numerically, we have to approximate the infinite Fourier expansions by finite ones, i.e. constrain the range of the reciprocal-lattice vectors $G = 2\pi n/a$ to $n = 0, \pm 1, \pm 2, \ldots, N$. For considered system, the range $N = 15$ gives satisfactory results for about six lowest frequency bands.

2 Width of the band gaps – dependence on B_0 and gaps between SC strips

In the discussion of Fig. 6, we briefly described how the width of the frequency gaps is modified with the external field B_0 and the distance between the SC strips d. To demonstrate the mentioned almost linear increase of the gap width with B_0 and the multiple gaps closing with d, we prepared Fig. S1.

The dependence of the width of the frequency gaps on the applied field, shown in Fig. S1(a), is close to linear. This is due to the fact that the profile of the stray field $\mathbf{B}_{sc}(x)$ scales linearly with the external field. This means that all of its Fourier components also scale linearly with B_0. Since the field $\mathbf{B}_{sc}(x)$ plays the role of a periodic coefficient in the linearized LL equations describing the SW dynamics in the considered MC, then the width of successive frequency gaps expressed by the corresponding Fourier coefficients should also have a linear dependence on B_0[2,3]. The linear scalability of the frequency gaps with an external field is a convenient feature of the MCs proposed in this work.

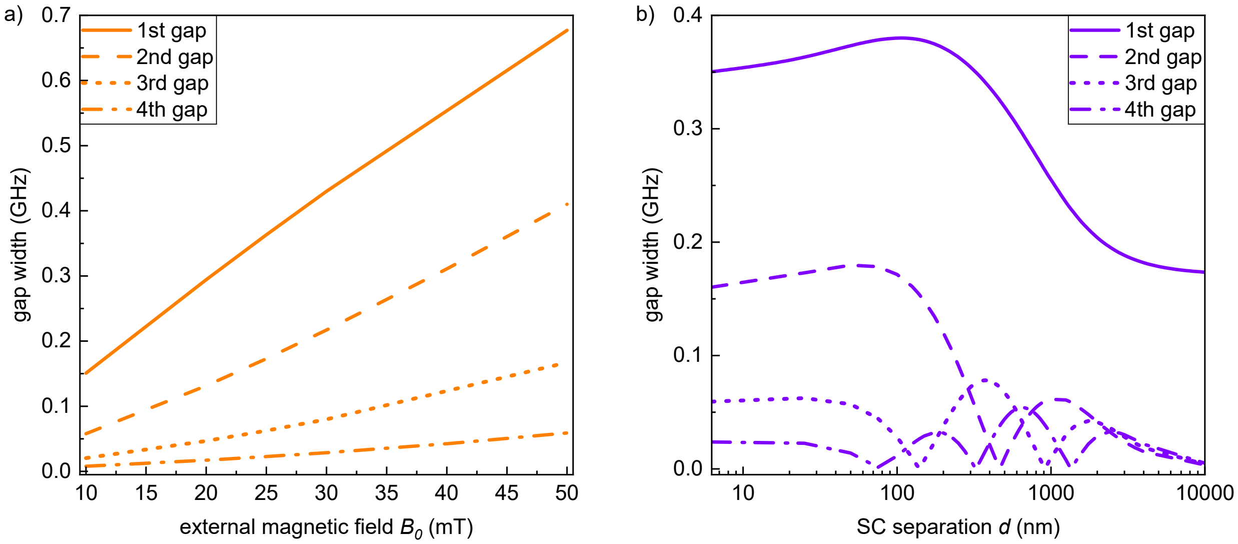

Figure S1. The width of the forbidden frequency gaps as a function of (a) external magnetic field and (b) superconducting strip spacing are shown. Parts of the drawing (a,b) correspond to Fig. 6(a) and Fig. 6(b), respectively. The orange (violet) numbers correspond to the gap number marked on Fig. 6(a) (Fig. 6(b)). On (b), the calculations have been performed for a separation range from 6.25 nm to 10 µm.

Fig. S1(b) presents the dependence of the width of successive gaps on the separation between SC strips. In the limit $d \to \infty$, the SW spectrum of MC is the same as the spectrum for the isolated well of the stray field. The magnonic bands for the frequencies below (above) the FMR frequency of the pristine FM layer will merge into a single level corresponding to bound states in the well (to continuous spectrum without gaps). Therefore in the limit $d \to \infty$, the width of the first gap, which separates the band laying below the FMR frequency, has finite width, while the higher gaps gradually disappear. This effect is clearly visible in Fig. S1(b).

On the other hand, for small separation between SC stripes d, we obtain unintuitive results. Even for small d, when the SC system seems to be almost continuous, the frequency gap stay wide open. It can be understood when we recall (see Fig. 2) that barriers of stray field become not only narrower with decreasing d but also higher. The competition between the width and height of the barrier determines the strength of the SW scattering the the width of frequency gaps.

In Fig. S1(b), it is easier to notice that the nth gap is closed $n-1$ times when bulk parameter d is swept over its whole range. It is explained by the effect of band crossing[4], known for wave excitations in periodic structures of different kinds, e.g. in photonic and phononic crystals.

3 Dynamical coupling between ferrimagnetic layer and superconducting pattern – FEM study

The comparative FEM calculations, demonstrating the impact of dynamic coupling between SC strips and FM layer, were performed in COMSOL Multiphysics in the following way. Initially, we solved the London equation assuming only the presence of the SC strip to calculate the static magnetic field produced by the superconductor in the absence of a ferromagnet. In these calculations, we assumed the finite London penetration depth $\lambda = 50$ nm. We considered the large vacuum domain above and

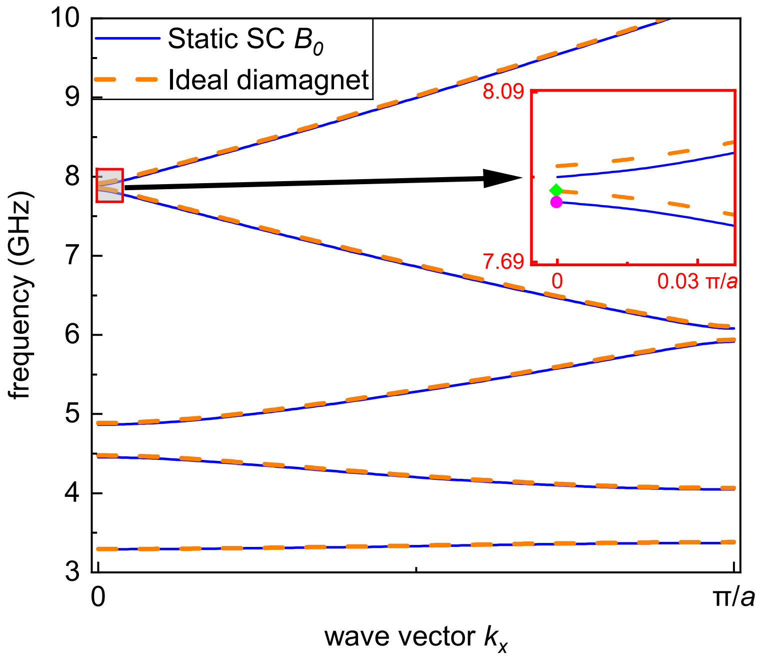

Figure S2. The FEM calculations of the SW dispersion relation for $B_0 = 50$ mT, $d = 25$ nm, $w = 400$ nm, without (blue lines) and with (orange lines) dynamic coupling between SC strips and FM layer. The coupling was introduced under the assumption of ideal diamagnetism of SC material where the field was completely expelled from the SC strips. The inset shows a tiny difference in the SW frequencies for both considered approaches. The magenta and green symbols mark the modes for which the maps of the dynamic demagnetizing field have been plotted in Fig. S3.

below the considered system which ensures that the field distribution will be properly calculated and Bloch boundary conditions on the edges of unit cell – dashed lines in Fig. S3. Then, we solved the LL equation and Gauss equation for magnetism within the magnetostatic approximation[5] to determine the SW dynamics, taking into account the static field produced by the SC strip calculated in the previous step. In this step, we performed two studies: (i) the SC strip was treated as a vacuum and did not produce any dynamic magnetic field, (ii) the SC strip was replaced with an ideal diamagnet for dynamic demagnetizing fields. The implementation of an ideal diamagnet is based on the boundary condition for the zeroing of the normal component of the magnetic field so that the magnetic field cannot penetrate the diamagnet. In COMSOL, this kind of boundary conditions are applied automatically by removing the volume of SC from the computational domain (see the white areas in Fig. S3(b)).

Fig. S2 shows that the dynamic coupling is very small for considered configuration, i.e. for the field B_0 applied at normal direction to the FM layer and SC strips.The dispersion branches for the hybrid system where only the static coupling was induced (blue line) are shifted down in frequency by a negligible amount when compared to the results where both the static coupling and the shielding of the dynamic demagnetization field were considered (orange lines).

To check that the effect of an ideal diamagnet was calculated properly, we show the profiles of h_x dynamic field produced by the fourth localized mode in the case without diamagnet in Fig. S3(a), and with diamagnet in Fig. S3(b). When the diamagnet is absent, the dynamic field penetrates the area where the SC should be present. When the diamagnet is present, the dynamic field is repelled from the diamagnet area, and its value increases inside the FM layer.

The mechanism of dynamic shielding of the magnetic field generated by SWs is well-known effect[6–8] in the case of conventional conductors. It is worth noting that dynamic shielding by superconductor in the Meissner state is more complicated[9,10] than static one, which is manifestation of ideal diamagnetism. The application of the $\mathbf{m}_{\text{eff}}(\mathbf{r},t) = -\mathbf{h}(\mathbf{r},t)$ relation valid for the static case in the $\lambda \to 0$ limit is, in general, not correct for dynamic shielding by superconductor. The relation between dynamic field, on which the superconductor is exposed $\mathbf{h}(\mathbf{r})e^{i\omega t}$, and its response, described by effective magnetization $\mathbf{m}_{\text{eff}}(\mathbf{r})e^{i\omega t}$, is given by the formula:[9,10]

$$\frac{1}{\lambda^2}\mathbf{h}(\mathbf{r}) = \Delta \mathbf{m}_{\text{eff}}(\mathbf{r}). \tag{S6}$$

Eq. S6 is derived from Faraday's law: $\nabla \times \mathbf{j}(\mathbf{r},t) = -\sigma/\mu_0\, \partial_t \mathbf{h}(\mathbf{r},t)$ and the relation between (superconducting) current $\mathbf{j}(\mathbf{r},t) = \mathbf{j}(\mathbf{r})e^{i\omega t}$ and (effective) magnetization: $\mathbf{j}(\mathbf{r},t) = \nabla \times \mathbf{m}_{\text{eff}}(\mathbf{r},t)$, taking the conductivity in the form typical for superconductors:

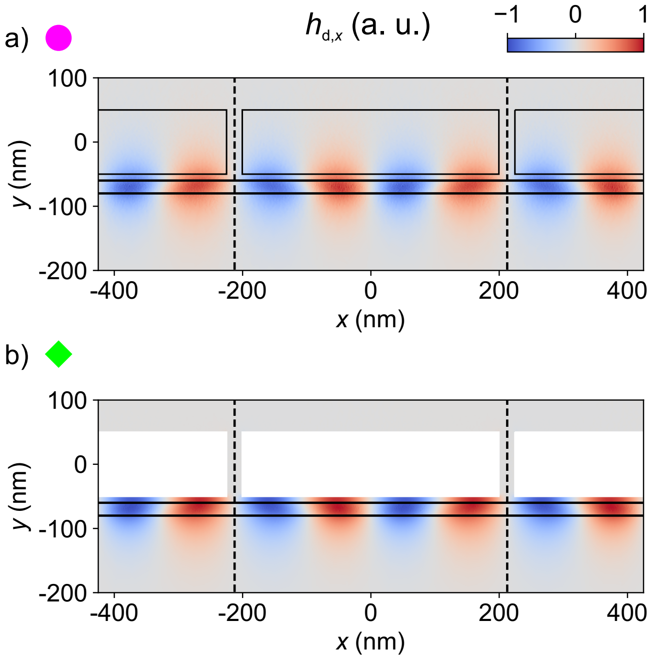

Figure S3. The maps of the real part of the in-plane component of the dynamic demagnetizing field $\Re[h_{d,x}(x,y)]$, for the mode marked by magenta and green in the inset of Fig. S2 – fourth mode at $k_x = 0$ in (a) absence and (b) presence of the dynamic coupling with SC strips approximated as ideal diamagnets. In both cases, the static component of the field produced by the SC strips is included. Two horizontal lines indicate the region of the FM layer. The black rectangles in (a) denote the domains of the SC strips, which expel only the static field but are transparent to the dynamic demagnetizing field produced by the propagating SWs. The white rectangles in (b) represent the regions of the SC strips where both static and dynamic components of the field are shielded.

$\sigma = i\,1/(\omega\mu_0\lambda^2)$. It is reasonable to assume that the field produced by the SW (see Fig. 5) and the resulting profile of the effective magnetization in the superconductor have the wavy pattern. Its wavelength $\lambda_{m_{\mathrm{eff}}}$ can be roughly estimated from Fig. 5. For example, for mode No. 3: $\lambda_{m_{\mathrm{eff}}} \approx 400$ nm and for modes Nos. 7 and 8, marked also in Fig. S2: $\lambda_{m_{\mathrm{eff}}} \approx 200$ nm. Under this assumption, we estimate from Eq. S6 the strength of dynamic shielding:

$$\mathbf{m}_{\mathrm{eff}}(\mathbf{r}) = -\frac{1}{(2\pi)^2}\left(\frac{\lambda_{m_{\mathrm{eff}}}}{\lambda}\right)^2 \mathbf{h}(\mathbf{r}). \qquad \text{(S7)}$$

The factor $-1/(2\pi)^2(\lambda_{m_{\mathrm{eff}}}/\lambda)^2$ takes the values about -1.6 and -0.4 for the mods No. 3 and Nos. 7 and 8, respectively. The FEM simulation were performed for the fixed value of this factor equal to -1. We think that even an enhancement according to Eq. S7 will not produce the noticeable change in the dispersion relation in Fig. S2.

References

1. Kaczér, J. & Murtinová, L. On the demagnetizing energy of periodic magnetic distributions. *Phys. Status Solidi A* **23**, 79–86, DOI: https://doi.org/10.1002/pssa.2210230108 (1974).
2. Tacchi, S. *et al.* Forbidden band gaps in the spin-wave spectrum of a two-dimensional bicomponent magnonic crystal. *Phys. Rev. Lett.* **109**, 137202, DOI: 10.1103/PhysRevLett.109.137202 (2012).
3. Yariv, A. & P. Yeh, O. *Optical Waves in Crystals* (Wiley- Interscience, New York, 2003).
4. Mieszczak, S. & Kłos, J. W. Interface modes in planar one-dimensional magnonic crystals. *Sci. Rep.* **12**, 11335, DOI: 10.1038/s41598-022-15328-x (2022).
5. Kharlan, J., Sobucki, K., Szulc, K., Memarzadeh, S. & Kłos, J. W. Spin-wave confinement in a hybrid superconductor-ferrimagnet nanostructure. *Phys. Rev. Appl.* **21**, 064007, DOI: 10.1103/PhysRevApplied.21.064007 (2024).
6. Gurevich, A. & Melkov, G. *Magnetization oscillations and waves* (CRC Press, London, 1996).

7. Mruczkiewicz, M. *et al.* Nonreciprocity of spin waves in metallized magnonic crystal. *New J. Phys.* **15**, 113023, DOI: 10.1088/1367-2630/15/11/113023 (2013).

8. Lisenkov, I. *et al.* Nonreciprocity of edge modes in 1D magnonic crystal. *J. Magn. Magn. Mater.* **378**, 313–319, DOI: https://doi.org/10.1016/j.jmmm.2014.10.073 (2015).

9. Borst, M. *et al.* Observation and control of hybrid spin-wave–Meissner-current transport modes. *Science* **382**, 430–434, DOI: 10.1126/science.adj7576 (2023).

10. Zhou, X.-H., Ye, X., Bai, L. & Yu, T. Giant enhancement of magnon transport by superconductor meissner screening. *Phys. Rev. B* **110**, L020404, DOI: 10.1103/PhysRevB.110.L020404 (2024).
